# Follow-up care after treatment for prostate cancer: protocol for an evaluation of a nurse-led supported self-management and remote surveillance programme

**DOI:** 10.1186/s12885-017-3643-4

**Published:** 2017-09-19

**Authors:** Jane Frankland, Hazel Brodie, Deborah Cooke, Claire Foster, Rebecca Foster, Heather Gage, Jake Jordan, Ines Mesa-Eguiagaray, Ruth Pickering, Alison Richardson

**Affiliations:** 1 Faculty of Health Sciences, University of Southampton, Highfield, Southampton, SO17 1BJ UK; 2 Faculty of Health and Medical Sciences, University of Surrey, Guildford, Surrey GU2 7XH UK; 3 School of Economics, University of Surrey, Guildford, Surrey GU2 7XH UK; 4 Medical Statistics Group, University of Southampton, Faculty of Medicine, Southampton General Hospital, Southampton, SO16 6YD UK; 5 Usher Institute of Population Health, University of Edinburgh, Teviot Place, Edinburgh, EH8 9AG UK; 6 University Hospital Southampton NHS Foundation Trust, Southampton General Hospital, Clinical Academic Facility, South Academic Block, Tremona Road, Southampton, SO16 6YD UK

**Keywords:** Prostate cancer, Follow-up care, Survivorship, Self-management support, Remote surveillance, Nurse-led

## Abstract

**Background:**

As more men survive a diagnosis of prostate cancer, alternative models of follow-up care that address men’s enduring unmet needs and are economical to deliver are needed. This paper describes the protocol for an ongoing evaluation of a nurse-led supported self-management and remote surveillance programme implemented within the secondary care setting.

**Methods/design:**

The evaluation is taking place within a real clinical setting, comparing the outcomes of men enrolled in the Programme with the outcomes of a pre-service change cohort of men, using a repeated measures design. Men are followed up at four and 8 months post recruitment on a number of outcomes, including quality of life, unmet need, psychological wellbeing and activation for self-management. An embedded health economic analysis and qualitative evaluation of implementation processes are being undertaken.

**Discussion:**

The evaluation will provide important information regarding the effectiveness, cost effectiveness and implementation of an integrated supported self-management follow-up care pathway within secondary care.

**Electronic supplementary material:**

The online version of this article (10.1186/s12885-017-3643-4) contains supplementary material, which is available to authorized users.

## Background

The number of people surviving after a diagnosis of cancer has increased dramatically in recent years, and is continuing to rise [1, 2]. For prostate cancer, an illustration of the 10 year survival rate is 84% in England and Wales [3] and 98% in the United States [4]. Cancer survivors are often left with challenging symptoms and side effects of treatment and with psychosocial concerns [5, 6]; specifically, prostate cancer survivors experience a range of physical symptoms, psychological and emotional difficulties and issues related to sexual function, such as impotence [7].This is presenting challenges for health care systems, in providing suitable follow-up care for those who have completed treatment [5, 8], and there is a need for more sustainable models of follow-up care which deal with capacity issues but also better address men’s survivorship needs.

In recent decades, a range of alternative follow-up care delivery models have been explored, including nurse led care, general/family practitioner led care, shared care, and patient initiated care [9]. Evidence suggests that these models are equivalent to the traditional clinic model in detection of recurrence and patient satisfaction [9]. In addition, a variety of psychosocial interventions have been developed to address men’s unmet survivorship needs, which appear to show some benefit for the men involved [10–12].

Within the last decade there has been recognition of the value of a self -management approach for cancer survivors [13]. In the United Kingdom (UK), the National Cancer Survivorship Initiative (NCSI) has promulgated an integrated, risk stratified and individualised model of cancer survivorship care, involving supported self-management and remote monitoring for the large proportion of cancer survivors who are at low risk of recurrence [14]. Such a model has recently been recommended for implementation in England by 2020, although a detailed model for prostate cancer remains to be developed and tested [15]. There is recognition of the international relevance of the principles of the model and its influence on programmes of care internationally [8].

Within this context a service improvement initiative, the TrueNTH Supported Self-Management and Follow-up Care programme (described henceforth as the Programme), has been funded in the United Kingdom by the Movember Foundation, in partnership with Prostate Cancer UK. The aim of the Programme is to implement a sustainable model of follow-up care within secondary care, based on the principles of risk stratification, supported self-management (SSM) and remote surveillance, to provide person-centred care through which to address men’s survivorship needs. The implementation of the Programme is accompanied by a comprehensive evaluation to assess effectiveness, cost effectiveness and implementation. This paper documents the evaluation protocol.

### The Programme

The Programme delivers personalised survivorship care through assessment of need, enhancement of men’s knowledge, skills and confidence to self-manage and easy access to advice and support. A stratified pathway approach [14] is employed, recognising that a proportion of post-treatment patients will be suitable for SSM.

The Programme was designed by a multi-disciplinary team, including urology health care professionals, experts in self-management techniques, and survivors of prostate cancer. The design team drew on previous work on the redesign of cancer follow-up care [14, 16] as well as the broad cancer survivorship and self-management literature. Urology and oncology teams at two National Health Service (NHS) Trusts were involved in the development of the clinical criteria used to judge suitability of men for the Programme and in piloting the Programme to assess feasibility and acceptability for both men and the clinical team.

**Fig. 1:**
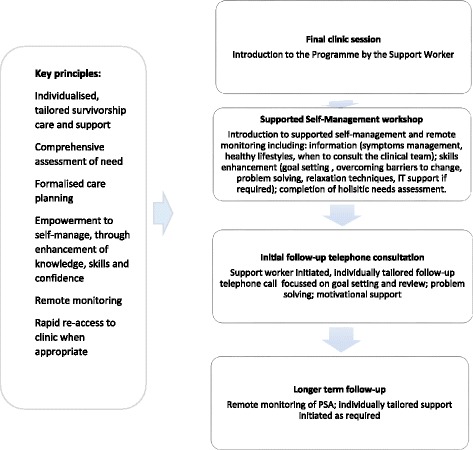
Components of the programme and underlying principles

Figure 1 details the components of the Programme and its underlying principles. The Programme is initially aimed at men being treated with radical prostatectomy, radiotherapy, or primary androgen deprivation therapy (PADT). Men are assessed for suitability at a post treatment clinic appointment with their consultant or Clinical Nurse Specialist (CNS), using clinical criteria including specified prostate specific antigen (PSA) levels for each treatment type, together with clinician assessment and discussion with the patient that they are functionally and emotionally suitable for SSM and remote surveillance. Men are first assessed for suitability from 6 weeks post radical prostatectomy and radiotherapy, or 3 months post commencement of PADT.

If a man is suitable for the Programme, this will be their last clinic appointment. Henceforth, they are monitored remotely by the specialist team, with a system of rapid re-access to clinic if indicated. There is the addition of a Support Worker role to the team, who is the mainstay of programme delivery, acting as coordinator of a man’s post treatment care and first point of contact for men who have queries or concerns, facilitating referrals to health and community resources. Men meet with the Support Worker at this point and are introduced to the Programme.

Soon after their entry into the Programme (ideally within 6 weeks), men attend a 4 h Supported Self-Management workshop, to prepare them for self-management and remote monitoring of their prostate cancer follow-up care, with a focus on living well after cancer treatment, promoting healthy lifestyles and setting personal health and wellbeing goals. Men complete a holistic needs assessment (HNA) during the session. Each workshop usually comprises between eight and 10 men and is facilitated by the CNS and Support Worker, who have been trained in workshop delivery skills and follow a facilitator manual. The workshops are based on principles of andragogy [17], Bandura’s social learning theory [18] and Adair’s action-centred leadership model [19].

The Support Worker initiates a follow-up telephone consultation after the workshop, to check that the man has grasped the main points put forward in the workshop and to answer any questions. A care plan is drawn up if appropriate. Contact with the man beyond this initial telephone call is negotiated individually, with the expectation that some men will need more contact and support for self-management than others.

Self-management and remote monitoring are facilitated by a bespoke Patient Online Service, which has both patient and health care professional facing functions. Men can access personal information such as treatment summaries and care plans, as well as validated sources of information to support self-management. They can submit their HNA and can have a two way conversation with a member of their clinical team via a secure system. The system prompts men when blood tests are due and men can see their PSA test results promptly. The health care team run virtual clinics through an electronic PSA tracking system which is interfaced with the Patient Online Service, reviewing PSA test results and HNAs, re-calling to clinic a man who has any indicators for concern.

## Methods/design

### Setting

The service re-design is being evaluated in four prostate cancer treatment centres within the NHS in England. Three sites were selected following an expression of interest process. Criteria for selection included enthusiasm of the clinical team, capability of IT departments to implement the proposed IT solution, and inclusion of hospitals in both urban and rural locations. The fourth site had previously been involved in development of the clinical criteria and piloting work, and was added as an evaluation site after 5 months of recruitment in order to boost numbers.

### Design

A mixed methods design is being implemented to assess the value of the Programme and to understand processes of implementation. The evaluation aims to: 1) assess the effectiveness of the Programme across key outcomes 2) assess the impact of the Programme on costs 3) assess the process of implementing the Programme, in order to identify any facilitating and inhibiting factors.

#### Evaluation of effectiveness

##### Design

The effects of the Programme on patient outcomes are being assessed by comparing the outcomes of men enrolled in the Programme with the outcomes of a pre-service change cohort of men, using a repeated measures design. The evaluation takes a pragmatic approach, testing the effectiveness of the Programme in a real clinical setting, allowing for clinical judgement in assessing men’s suitability for the new service and for flexibility in service delivery [20].

##### Comparator group

The comparator group is a pre-service change group of men, recruited from the cohort of men in prostate cancer follow-up care at the four sites during the period immediately prior to the introduction of the Programme. The comparator group men receive their hospital’s standard follow-up care (standard care) as it was before the service change; this is either clinic based follow up with a urological surgeon, oncologist or CNS, or telephone follow up with a CNS.

Where capacity allows, men in the comparator group are migrated to the new service once they have completed their final study questionnaire.

##### Eligibility

Men are eligible to enter the evaluation if they are: i) within 3 years of completion of radical prostatectomy/radiotherapy or within 3 years of commencement of PADT ii) are 18 years of age or older and iii) have adequate English language ability to complete study questionnaires. Men who are unable to give informed consent and men participating in clinical trials which require face-to-face contact are excluded.

##### Data collection

**Fig. 2:**
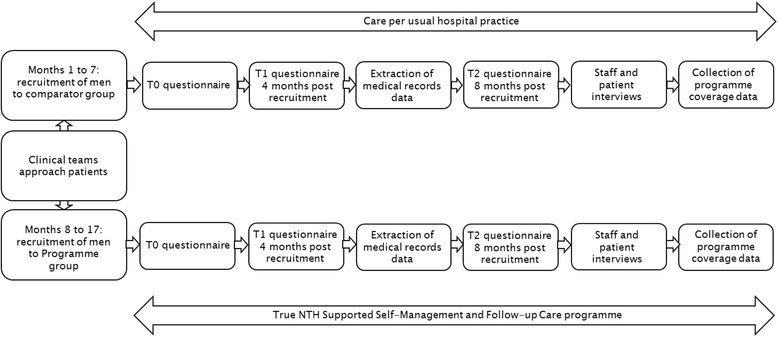
Study flow

Data are collected by postal questionnaire at recruitment (T0), then 4 months (T1) and 8 months post-recruitment (T2) (see Fig. 2). Clinical and treatment data (cancer stage, grade, date of diagnosis, and treatment received) are collected from medical records. If a participant dies during their involvement with the study, it is ascertained whether the death was due to prostate cancer.

##### Recruitment

Men were recruited to the comparator group between September 2014 and June 2015, and to the Programme group between April 2015 and February 2016, with data collection completed in December 2016.

Men attending a clinic appointment who met the eligibility criteria for the evaluation were initially approached by clinical staff or a research nurse, to introduce the evaluation and to ask for consent for their contact details to be passed to the research team. Men who consented to contact were sent, by post, an introductory letter, a patient information sheet, consent form, baseline questionnaire and a freepost envelope for return of the completed documents.

##### Outcome measures

The evaluation is comparing the Programme with standard care across a number of different outcomes; a series of validated patient reported outcome measures are being used (see Table 1), to reflect multiple outcomes of interest relevant to the theoretical model underpinning the Programme [21].

**Table 1:** Outcome measures

Outcome measures at T0, T1 and T2			
Description of outcome	Measure used	Description of measure	Psychometric/biometric properties of measure
Unmet need (primary outcome)	Cancer Survivors’ Unmet Needs Measure, (CaSUN) [22]	35 questions regarding unmet need in 5 domains of existential survivorship; comprehensive cancer care; information; quality of life; relationships. Additional 6 questions about positive life changes and space for free comments The unmet need questions can be scored as individual items or as scores of unmet and total need, and/or strength of need.	Due to reported difficulty with the five point response format used by the CaSUN, this study has followed others in using a simplified four point response format [35]
Health status	EQ5D-5 L [24]	5 questions addressing mobility, self-care, daily functional status, pain and/or discomfort, anxiety and/or depression; 5 point Likert scale; converts to single index value for calculation of quality-adjusted life years; utility estimates have been provided for cancer patients [36].	There is support for the scale’s reliability and validity when used with cancer patients [36]
Prostate cancer health related quality of life	Expanded Prostate Cancer Index Composite Short Form (EPIC-26) [37]	26 questions in 5 domains of urinary incontinence; urinary irritative/obstructive; bowel; sexual; and hormonal; 4 and 5 item Likert scales; individual items are scored as a total on a 0–100 scale.	Good internal consistency (Cronbach’s alpha ≥ 0.70 for each domain) and test-retest reliability (*r* ≥ 0.69 for each domain) [38].
General cancer related quality of life	Functional Assessment of Cancer Therapy Scale - General (FACT-G) [39]	28 questions in 4 domains of physical wellbeing; social/family wellbeing; emotional wellbeing; functional wellbeing. 5 item Likert from ‘not at all’ to ‘very much’; provides domain scores and a total score.	Reliability and validity have been shown to be high [39]
Psychological well being	General Health Questionnaire (GHQ-12) [40]	Measure of current mental health using 12 questions and 4 point Likert scale that provides an overall total score.	The GHQ-12 has been extensively evaluated and shows good validity and reliability [41].
Activation to self-manage	Patient Activation Measure (PAM®) [42]	The short form comprises 13 questions answered on 5 point scale, from ‘disagree strongly’ to ‘agree strongly’ plus N/A; scores from a minimum of 10 responses into one of 4 levels of activation.	The short form version has been shown to be reliable and valid [43]
Worry about cancer recurrence	Adaptation of the Worry of Cancer Scale [44]	2 questions to measure frequency and degree of intrusiveness of worry about cancer recurrence; Likert scales of 0–10 and 0–4; calculates score of 0–20	No psychometric data for the modified version, but the original scale performed well [44]
Health behaviours - diet	Adaptation of the Fruit and Vegetable Screening Measure for Adolescents [45]	Rating fruit and vegetable consumption in a typical day, as two separate items; response adapted to include 5 or more portions, to be in line with current guidance	The measure has shown to be reliable and valid [45]
Health behaviours – physical activity	Leisure Time Exercise Questionnaire [46]	Measure of leisure time physical activity as mild, moderate and strenuous activity, plus activity to work up a sweat as number of units of 15 plus minutes per week. A total score is calculated, which can be used to classify respondents as active, moderately active and insufficiently active [47]	Reliability and validity of the measure has been shown [46] The classification system has been validated or use with cancer survivors [48]
Health behaviours - smoking	Developed for this study, drawing on measurement of these items in health promotion work.	1 question to measure current smoking behaviour	
Health behaviours - alcohol	Developed for this study, drawing on measurement of these items in health promotion work.	1 question to record frequency of current alcohol consumption	
Outcome measures at T1 and T2			
Health service use	Developed for this study, based on a previous evaluation at the University of Southampton [16]	15 questions about contact with health and community services for prostate cancer related issues and patient costs related to prostate cancer	
Satisfaction with follow-up care	Developed for a previous evaluation at the University of Southampton [16]	11 questions and space for free text comments regarding experience and acceptability of follow-up care	
Other variables			
Demographic characteristics	Date of birth, ethnicity, marital status, level of education, employment status, housing status, caring responsibilities, computer use.		

Measures of general health status, physical symptoms, cancer specific quality of life, unmet needs, psychological wellbeing, worry about cancer recurrence, activation for self-management, and general health behaviours are being collected at T0, T1 and T2 time points. In addition, questions about health service use and satisfaction with follow-up care are included in the T1 and T2 questionnaires. Socio-demographic characteristics are also collected. The primary outcome is unmet need, measured with using the Cancer Survivors Unmet Needs measure [22].

##### Sample size calculation

The sample size was calculated to achieve at least 90% power in two sided tests for variables which achieve a moderate intervention effect (0.3 or larger). This would require a sample of 235 participants per group and we therefore aimed to recruit around 300 men to each arm of the study, allowing for 20% being lost to follow-up by the 8 month assessment.

##### Data analysis

Data analysis will follow a pre-specified data analysis plan. Analyses will be conducted on an ‘intention to treat’ basis. We will first describe the baseline characteristics of participants at time T0 within each group and will compare clinical and demographic characteristics and outcome measure scores of the two groups. The same comparisons will be made with baseline data between those continuing to four and 8 month follow-up and those lost to attrition.

A regression analysis will be conducted for each of the outcome measures at the 4 month and 8 month time points separately, controlling for study site, the outcome in question if available at T0, and baseline co-variates including age, type of treatment, educational attainment, time since diagnosis, domestic status, co-morbidity, employment status, and ethnic status. If other factors differ between groups at baseline, additional controlled analyses will be carried out.

We will also estimate a mixed model including outcome at four and 8 months simultaneously, including an interaction to give separate estimates of the Programme versus standard care comparison at each follow-up point.

Finally, we will investigate whether there are any distinct subgroups for whom the intervention is effective. Guided by existing literature to indicate likely subgroup effects, separate regression analyses will be conducted for older versus younger men, men with and without co-morbidities, and men with higher and lower levels of deprivation. We will address subgroup analyses within the context of regression modelling (either linear or logistic). Interaction terms between each specified dichotomous factor (indicating subgroups) and the Programme versus standard care factor will be examined. Where interaction terms achieve statistical significance (at the 5% level), we will examine separate Programme versus comparator group estimates for each subgroup. All subgroups analyses will be considered exploratory in nature.

#### Economic evaluation

##### Design

The economic evaluation will compare costs and health outcomes of men in the Programme and the comparator group of men in standard care. The primary analysis will be from a health service perspective, with a secondary analysis from the patient perspective. A cost-consequence analysis will be conducted using the full range of outcome measures.

##### Data collection

Costs of the workshop and the Patient Online System will be sourced from providers. The IT costs will be annuitized over a plausible useful lifespan assuming optimal utilisation to provide a realistic cost per patient. The nurse time for monitoring and follow up using the surveillance system will be sourced from observation of a small sample of the staff performing the activity in situ. This resource use will be priced using unit costs based on national tariffs [23] or from site finance managers.

Self-reported service use data is collected in the four and 8 month questionnaires. This captures health, social and voluntary service use, including contacts with the General Practitioner, Practice Nurse, District Nurse, social worker, physiotherapist, dietician, psychologist, complementary therapies, outpatient appointments, and hospital stays. Data on routine clinic appointments and telephone contact with the urology team will be sought from site staff. Service use will be costed using national tariffs [23]. All participants will also be asked to report out-of-pocket expenses on travel to any appointments and on prostate cancer related care products and medications, and any time spent by informal carers in supporting the patient. The primary outcome measure for the economic evaluation is the EQ-5D-5 L [24] collected at all three data collection points.

##### Analysis

The average cost per patient will be calculated for both Programme and comparator groups. For the Programme group, this will comprise the workshop, the Patient Online System and associated nurse time, and the costs of all health and social care. The costs for the comparator group will comprise the cost of standard care and all other health and social care. The individual EQ-5D-5 L survey results will be used to estimate patient utility at each time point. These will be integrated over the 8 month follow up using the area under the curve method to calculate accrued quality-adjusted life years (QALYs) for each participant. Average QALY differences between groups will be estimated using ordinary least squares regression, controlling for differences in baseline utility. White adjusted standard errors will be used to account for unobserved heterogeneity.

The total cost per patient and total QALYs per patient will be compared between the Programme and comparator groups using the incremental cost effectiveness ratio (ICER). Uncertainty will be handled non-parametrically using bootstrap resampling with replacement [25]. Deterministic sensitivity analysis will be conducted around main drivers of cost, and to allow for specific uncertainties around estimated unit costs. Exploratory multivariate regression analysis will be employed to assess the relationship between health and cost outcomes and other sociodemographic data. The cost consequences analysis will compare the two service delivery models across the full range of outcomes.

#### Assessing implementation

##### Design

The aim of this component of the evaluation is to assess the process of implementation of the Programme and to identify any barriers and facilitators to implementation. Data are being collected though semi-structured interviews with staff involved in service delivery or management, and with a subsample of men taking part in the questionnaire study. Normalisation Process Theory (NPT) [26] is being used to sensitise the interviews to factors which may help or hinder the embedding of the Programme at the sites.

##### Data collection

Semi-structured interviews are completed with a sample of up to 10 staff from each of the four study sites. Both clinical and managerial staff involved with the Programme were identified through discussion with the lead clinician and lead nurse at each site. These interviews are conducted by telephone and focus on experiences of implementing the Programme.

Similarly, interviews are being conducted with a maximum of 12 men from each of the four study sites, to include men who have experienced standard care (maximum of four men per site) and men enrolled in the Programme (maximum of eight men per site). The interview guides for both patients and staff are provided in Additional file 1. Men were purposively selected from those who indicated on their baseline questionnaire that they were happy to be contacted regarding an interview. The sample was selected to take account of age, type of cancer treatment, time since diagnosis and computer usage. The interviews focus on men’s experience of follow-up care and the post treatment period, and take place by telephone or face-to-face according to preference.

##### Analysis

Each audio-recording is transcribed verbatim and is checked against the recording. The analysis is taking a team approach. The initial coding frame was developed through a process of independent coding and subsequent discussion of a number of transcripts by at least two of the study team. The development of codes took both a deductive and inductive approach, having an initial focus on the research question, NPT constructs and the underlying theory of change, but also being open to codes that emerge from the data. The reliability of the individual coders was established over a number of transcripts and then the coding frame will be applied to remaining transcripts. Analysis will involve the constant comparison of data and close attention to deviant cases. Regular evaluation team meetings will be held to reach consensus on themes and findings.

##### Ethics and reporting

The study received ethical approval from the National Research Ethics Service, East of England – Cambridge South (reference number 11/EE/1021), research governance approval from the individual NHS Trusts involved with the study, and has been adopted by the National Institute of Health Research Clinical Research Network (ID 17238). Study reporting will follow appropriate guidelines [27–29].

## Discussion

The TrueNTH Supported Self-Management and Follow-up care Programme implements an integrated supported self-management and remote monitoring model of follow-up care within secondary care. The Programme aims to address contemporary problems with clinic capacity, but also to offer men a more tailored follow-up care experience that addresses their individual needs. While other alternatives to the clinic based model of post treatment care continue to be tested (for example, [30–32]), this Programme offers an alternative which maintains specialist oversight and input.

The Programme was funded as a service improvement initiative with accompanying evaluation. The evaluation takes a pragmatic approach [20], seeking to answer questions about effectiveness of the model within an everyday clinical setting, and also to provide information on the practicalities of implementing and sustaining such a model. Such information should provide relevant and useable conclusions for local decision makers considering implementation of a similar model in their setting [20].

There are a number of limitations to the study design. First, the use of a non-randomised comparator group represents a reduction in the ability of the study to attribute outcomes to the Programme, though the inclusion of a baseline measurement helps to address this issue, allowing for statistical adjustment of known confounders [33]. Second, men will be followed up for a period of 8 months, meaning that the study will only be able to comment on outcomes over this time period and not over a longer term [21].

Within the context of the rising number of cancer survivors, alternatives to resource intensive, medically focussed models of follow-up care are required, and supported self-management has been suggested as beneficial for cancer survivors with stable disease [34]. This evaluation of a supported self-management programme for men with prostate cancer will provide useful information for management of this particular group, but will also add to the literature on alternatives to clinic based follow-up care which will be of relevance to other groups of cancer survivors across the globe.

## Additional file

Additional file 1: Interview guides for patient and staff interviews. (DOCX 37 kb)

## Abbreviations

CNS: Clinical Nurse Specialist
HNA: Holistic Needs Assessment
ICER: Incremental cost effectiveness ratio
IT: Information technology
NCSI: National Cancer Survivorship Initiative
NHS: National Health Service
PADT: Primary androgen deprivation therapy
PSA: Prostate specific antigen
QALY: Quality-adjusted life year
SSM: Supported self-management
T0: Baseline assessment point
T1: 4 month assessment point
T2: 8 month assessment point
