# Supplementary material for: Follow-up care after treatment for prostate cancer: protocol for an evaluation of a nurse-led supported self-management and remote surveillance programme
Source: BMC Cancer. 2017 Sep 19;17:656. doi: 10.1186/s12885-017-3643-4 (PMC5606080; doi:10.1186/s12885-017-3643-4)
Supplement: Additional file 1: — Interview guides for patient and staff interviews. (DOCX 37 kb) [file 12885_2017_3643_MOESM1_ESM.docx]

**Follow-up care after treatment for prostate cancer: protocol for an evaluation of a nurse-led supported self-management and remote surveillance programme.**

**Interview schedules**

**Patient interview schedule - Programme group**

*Reminder of purpose of the study, re-address informed consent, explain interview process, agree recording of interview*

**Introduction**

To start with, could you tell me a little bit about yourself: the treatment you’ve had; how you’ve been since your treatment; whether you are working and, if retired, what work you did; who you live with?

- Could you tell me what is currently happening with the hospital, following your treatment for prostate cancer?

1. **Moving into the Programme**

- Who introduced the Programme to you?
- Had you just finished treatment or had you been receiving clinic based or telephone follow-up care before this?
- Were you expecting your care/treatment to change at this point?
- Did it make sense to you?
- Did you understand what was being asked of you?
- Did you feel you were given a choice about it?
- At this point in time did you have any concerns about it?
- At this point in time did you see any benefits of taking part?
  - Did you discuss the Programme with anybody (like family or friends)? What did they think of it?
  - Have you been part of any other similar programmes of care, perhaps for another health condition? What and for how long?

1. **Views of the Programme**

*Next, I would like to talk about how you have found the different parts of the Programme*

**Workshop**

- - How did you feel about the idea of the workshop before you attended?
- What did you think of the workshop when you did attend? What did you like? What didn’t you like? What did you find useful? What did you find less useful?
- How did you find being with other men with a similar experience?
- How was it being in an all-male group?
- Did you discuss anything you hadn’t been able to discuss with someone previously? How and why do you think the workshop enabled you to discuss these?
- Do you remember doing the good planning section – talking about how you might plan any changes you wanted to make? What did you think about this? How did that go?
- At the end of the workshop, how did you feel about dealing with your prostate cancer follow up care?
- And at the end of the workshop, how did you feel about dealing with any side effects of your treatment?
  - Did you have any concerns at this point about anything that you were being asked to do? E.g. Computer skills? Understanding of HNA? Able to telephone hospital?
  - Did you discuss any aspects of the workshop with family or friends afterwards? What sort of things? How did that go?
  - Did you go back to staff at the hospital with any questions about the workshop or what you were being asked to do? What sort of questions? How did that go?
  - Do you keep in touch with any of the men that attended the workshop with you? Is that helpful?

If didn’t attend workshop

- Why didn’t you attend?

**Handbook**

*Do you remember that you were given a handbook at the workshop?*

- Have you found that useful*?*
  - If **no**: why not?
  - If **yes**, what parts do you use and why? How are these useful to you?

**Computer system**

- Do you use the computer system?

If YES

- How often/when do you use it?
- What sorts of things do you use it for? To look at PSA results? How useful is it to you to be able to get your results in this way? Messaging? Who/what? Finding information? What sorts of information/where?
- How do you find using the computer system?
- What do you like about it/not like about it?
- Do you use a computer at home for other things? What sorts of things/how do you find it?
- Does anyone help you to use the computer system or use it on your behalf to access information on your follow up care?

If NO

- Why do you not use it?
- Do you have paper communication with the hospital instead (eg for PSA results)?
- How do you find this?

**Ongoing support**

*Do you remember having a telephone appointment/call with the support worker after the workshop?*

- How did you find that? Was it useful?
- Did you have any further telephone appointments/calls after that?
- How did you find that?
- Did anything happen as a result of these calls?

**PSA tests**

- How often do you have a PSA test now?
- And how does this happen?
- How do you find that process?
- How do you feel about waiting for your PSA results?
- If they were in clinic follow up before the Programme, is getting the results quicker a good thing?

**Holistic Needs Assessment**

- Do you remember filling out a Holistic Needs Assessment at the workshop?
- How was that? Did you find it useful? Did anything happen as a result?
- Have you filled out a Holistic Needs Assessment since the workshop?
- **If YES:** How often/when do you do this? Probe: each time you have a PSA test/ only if you have a problem?
- Has filling it out been useful to you?
- Have you ever been contacted by the Programme staff as a result of filling out the form? Can you tell me more about what happened?
- **If NO**: Why have you not filled it out?

1. **Seeking help**

*I would now like to talk about where you would get help if you had a question or problem related to your prostate cancer.*

- Have you actually had any questions or needed some help since you have been on the Programme? Explore: what they did and how it went; why they chose to do that; what sort of response they got; were they happy with how it went; would they do the same again?
- If you had a question or needed some help for anything related to your prostate cancer in the future, where would you go to get that help? Explore who and how. Why would you choose that source of help?
- What sorts of problems or concerns to do with your prostate cancer do you think are appropriate to ask the Programme staff about?
- Ask about each of :
- Physical problems
- Side effects of treatment
- Emotional concerns
- Relationship concerns
- Practical concerns
- If not appropriate to ask hospital, then why not, and where would you go for help?
- How do you feel about approaching the Programme staff at the hospital?
- If you did ask the Programme staff, what sort of response do think you would get?

1. **Looking after yourself**

*Could we now talk about anything you do yourself to look after yourself after your prostate cancer treatment?*

- In between your PSA tests, do you do anything to look after yourself following your prostate cancer treatment? What sorts of things? (eg pelvic floor exercises, relaxation) How does that go? What prompted you to do this?
  - In between your PSA tests, do you do anything for yourself to help with any ongoing issues/problems you have had as a result of your treatment for prostate cancer? What sort of things and how does that go? What prompted you to do this?
  - Have you made any changes to any everyday behaviours related to your health since you went onto the Programme? Probe: such as diet, exercise, alcohol or smoking. What prompted you to do this?
- Is there anything that you learnt about at the workshop that you have continued to do? What sort of things and how does that go?
- Do you have any help or support from someone else in doing any of this?

1. **Reflection on the Programme**

*I would now like to talk to you about your overall views of the Programme*

- Overall, how has the Programme been for you so far? What do you think about it? How do you feel about it? What do you like about it? What do you not like about it?
- Do you feel prepared to manage your follow up care? Do you think have all the knowledge, information, help, support that you need?
- Do you feel prepared to look after yourself following your prostate cancer treatment? Do you think have all the knowledge, information, help, support that you need?
- Is there anything that would improve your experience of follow-up care?
- Overall, do you see any benefits from this programme for
  - the hospital
  - for people like you
- **If have experience of clinic follow up care:** as you were in clinic based follow up care before moving to the Programme, how do the two compare for you?
- If you were meeting someone who was finishing treatment for prostate cancer and starting on the Programme, would there be any advice you would give to them?
- Is there anything else that I haven’t asked that you want to add?

**Close of interview**

**Patient interview schedule – Comparator group**

*Reminder of purpose of the study, re-address informed consent, explain interview process, agree recording of interview*

**Introduction**

To start with, could you tell me a little bit about yourself: the treatment you’ve had; how you’ve been since your treatment; whether you are working and, if retired, what work you did; who you live with?

- Could you tell me what is currently happening with the hospital, following your treatment for prostate cancer?

1. **Experience of follow up care**

- Can you describe to me a bit more about your follow up care appointments with the hospital?
- And with the PSA tests: How do you get the results? How long is it for you to get the results? How do you feel about the wait for those?
- What do you think about the appointments?
- Have you ever raised any problems you are having related to your prostate cancer at these appointments? What sorts of things? How did that go?
- What sorts of things would you feel you could ask about (physical problems, side effects, emotional/mental health, relationship concerns, practical concerns)?
- Have you ever discussed how to look after yourself after prostate cancer treatment at these appointments? (side effects/healthy lifestyle?)

1. **Seeking help**

*I would now like to talk about where you would get help if you had a question or problem related to your prostate cancer in the time between your hospital appointments.*

- Have you actually had any questions or needed some help in the time between your hospital appointments? What did you do and how did that go? Why did you chose to do that? Were you happy with how it went? Would you do the same again?
- If you had a question or needed some help for anything related to your prostate cancer in the future, where would you go to get that help?
  - Ask about each of :
    - Physical problems
    - Side effects of treatment
    - Emotional concerns
    - Relationship concerns
    - Practical concerns

1. **Looking after yourself**

- In between your hospital appointments/PSA tests, do you do anything to look after yourself following your prostate cancer treatment?
- Do you do anything to deal with any ongoing issues/problems you have had as a result of your treatment for prostate cancer. What sorts of things? (eg pelvic floor exercises, relaxation) How does that go? What prompted you to do this?
- Have you made any changes to any everyday behaviours related to your health since you went onto follow up care? Such as diet, exercise, alcohol or smoking. What prompted you to do this? Do you have any help or support from someone else in doing any of this?

1. **Reflection on your follow up care**

*I would now like to talk to you about your overall views of your follow up care*

- Overall, how has your follow up care been for you so far? What do you think about it? How do you feel about it? What do you like about it? What do you not like about it?
- Do you feel prepared for your follow up care? Do you think have all the knowledge, information, help, support that you need?
- Do you feel prepared to look after yourself following your prostate cancer treatment? Do you have all the knowledge, information, help, support that you need?
- Is there anything that would improve your experience of clinic follow-up care?
- If you were meeting someone who was finishing treatment for prostate cancer and starting on follow up care would there be any advice you would give to them?
- Is there anything else that I haven’t asked that you want to add?

**Close of interview**

**Staff interview schedule**

*Interviews with staff cover a variety of roles and involvement with the Programme (for example project leads, lead implementers, wider team, related managerial and support). The questions are used and adapted as appropriate for each interviewee’s role.*

**Interview purpose**

*Reminder of purpose of the study, re-address informed consent, explain interview process, agree recording of interview*

**1) Regarding person and role**

- - Could you begin by telling me a bit about your professional background?
  - Could you briefly describe your current role in the urology team and involvement with the Programme?
  - What challenges are the urology team facing in relation to treatment and follow up of prostate cancer patients?

**2) Regarding introduction of the Programme**

- What was your role in bringing the Programme to the service? When did you become involved?
- When the Programme was introduced, what benefits did you think the Programme would bring? For the Trust; service; self; patients
- When the Programme was introduced, did you have any concerns about the programme and its implementation?
- Were you involved in gaining agreement at management level? Could you describe the processes involved? Were there any issues or challenges?
- Were you involved in introducing the Programme to colleagues or other members of the team? Were there any issues/challenges related to this? Is everyone on board? What helped with this?

**3) Regarding implementation of the Programme**

**Support worker role (role not the individual)**

- How has it been having the new support worker role within the team?
- How has this new role fitted into the team?
- Have there been any challenges with this role?
- What would you change, if anything, about this role? E.g. grade, type of skills required, taking on some of the work of the CNS?
- (**For support worker**) How have you found the role? Have there been any challenges in enacting the role?

**Screening and referral**

- What role have you played in screening men for eligibility, and referring them to the Programme?
- Roughly how many patients have you personally referred onto the programme?
- What challenges, if any, have you encountered in the screening and referral process?
- How did you find use of the eligibility tools and protocols? Did you feel that the criteria identified the right patients?
- How have you found patients’ reactions? Has that made a difference to your decision at any time? How does their reaction compare to men going into traditional follow up care

**Workshops**

- Have there been any challenges for you in delivering the workshops? (probe: time management with other work, skills, training)
- What has helped with delivering workshops?
- Do you feel confident that the men who have attended the workshops have understood supported self-management and remote monitoring and what is required of them?

**Patient on-line service**

- Have there been any challenges with the patient online service? Explore set up and use
- Has anything in particular helped in delivering the patient on-line service?

**Holistic Needs Assessment**

- Have there been any issues/challenges in delivering the Holistic Needs Assessment? (getting men to do it, knowledge, skills, resources , fitting it into work schedule)
- Has anything helped with delivering the Holistic Needs Assessment?

**Remote monitoring and virtual clinics?**

- What challenges have you been experiencing with the remote monitoring and virtual clinics?
- What has been going well with these clinics? Has anything helped?

**Dealing with queries from men on the programme**

- Have you dealt with any queries from men/or their representatives on the programme? What sorts of things have they been asking?
- Have there been any challenges in dealing with these? Has anything helped?
- **Relationship with patient group**
- What sort of relationship do you have with the men on the Programme?
- Does this relationship differ to men on clinic follow up?
- How are these relationships built up and established?
- Is this relationship valuable to the success of the Programme?

**Project management**

- Were there any challenges in leading and managing the implementation of the Programme?
- What aspects of support and processes provided by the Programme project-manager role would be vital in the future take-up of the intervention?

**4) Regarding evaluation of the Programme**

- Have you noticed any impact of the Programme on your work and on the team?
- Have you seen any benefits of the Programme for your work/for patients/for the service/for the Trust?
- Are there any downsides for you, patients, the service, the Trust?
- Have you/the team needed new knowledge, skills, ways of working to implement the Programme? Have you/they been provided with adequate access to training for these?
- What sorts of skills do patients need for self-management and remote monitoring? Do you think Programme men have these now?
- How does the safety and quality of service provided to men on the programme compare to that of men in clinic based care?
- With the benefit of hindsight, would you do anything differently in relation to your involvement in the programme?

**5) Regarding future of the Programme**

- What is the future of the Programme in your hospital trust? Is there strong motivation within the trust to keep going with the programme after the pilot has finished?
- What processes will you need to undertake to ensure that the Programme continues?
- Do you anticipate any parts of the delivery of the Programme changing in the future?

**That’s the end of our questions. Is there anything else that you would like to add, that we haven’t covered with these questions?**

**Close of interview**
